# Genome-wide analysis and prediction of genes involved in the biosynthesis of polysaccharides and bioactive secondary metabolites in high-temperature-tolerant wild *Flammulina filiformis*

**DOI:** 10.1186/s12864-020-07108-6

**Published:** 2020-10-17

**Authors:** Juan Chen, Jia-Mei Li, Yan-Jing Tang, Ke Ma, Bing Li, Xu Zeng, Xiao-Bin Liu, Yang Li, Zhu-Liang Yang, Wei-Nan Xu, Bao-Gui Xie, Hong-Wei Liu, Shun-Xing Guo

**Affiliations:** 1 Key Laboratory of Bioactive Substances and Resource Utilization of Chinese Herbal Medicine, Ministry of Education, Institute of Medicinal Plant Development, Chinese Academy of Medical Sciences and Peking Union Medical College, Beijing, P. R. China; 2 State Key Laboratory of Mycology, Institute of Microbiology, Chinese Academy of Sciences, Beijing, P. R. China; 3 Key Laboratory for Plant Diversity and Biogeography of East Asia, Kunming Institute of Botany, Chinese Academy of Sciences, Kunming, P. R. China; 4 Mycological Research Center, College of Life Sciences, Fujian Agriculture and Forestry University, Fuzhou, P. R. China

**Keywords:** Edible mushroom, Gene cluster, Gene expression, Polysaccharides, Sesquiterpene, High-temperature-tolerance

## Abstract

**Background:**

*Flammulina filiformis* (previously known as Asian *F. velutipes*) is a popular commercial edible mushroom. Many bioactive compounds with medicinal effects, such as polysaccharides and sesquiterpenoids, have been isolated and identified from *F. filiformis*, but their biosynthesis and regulation at the molecular level remains unclear. In this study, we sequenced the genome of the wild strain *F. filiformis* Liu355, predicted its biosynthetic gene clusters (BGCs) and profiled the expression of these genes in wild and cultivar strains and in different developmental stages of the wild *F. filiformis* strain by a comparative transcriptomic analysis.

**Results:**

We found that the genome of the *F. filiformis* was 35.01 Mb in length and harbored 10,396 gene models. Thirteen putative terpenoid gene clusters were predicted and 12 sesquiterpene synthase genes belonging to four different groups and two type I polyketide synthase gene clusters were identified in the *F. filiformis* genome. The number of genes related to terpenoid biosynthesis was higher in the wild strain (119 genes) than in the cultivar strain (81 genes). Most terpenoid biosynthesis genes were upregulated in the primordium and fruiting body of the wild strain, while the polyketide synthase genes were generally upregulated in the mycelium of the wild strain. Moreover, genes encoding UDP-glucose pyrophosphorylase and UDP-glucose dehydrogenase, which are involved in polysaccharide biosynthesis, had relatively high transcript levels both in the mycelium and fruiting body of the wild *F. filiformis* strain*.*

**Conclusions:**

*F. filiformis* is enriched in a number of gene clusters involved in the biosynthesis of polysaccharides and terpenoid bioactive compounds and these genes usually display differential expression between wild and cultivar strains, even in different developmental stages. This study expands our knowledge of the biology of *F. filiformis* and provides valuable data for elucidating the regulation of secondary metabolites in this unique *F. filiformis* strain.

## Background

*Flammulina filiformis*, also known as enokitake, winter mushroom or golden needling mushroom, is a species endemic to Asia and belongs to the family Physalacriaceae, Agaricales [1]. Previously, *F. filiformis* from eastern Asia was regarded as Asian *F. velutipes* or *F. velutipes* var. *filiformis,*but recently phylogenetic results based on multi-gene markers and morphological comparisons demonstrated that “*F. velutipes*” in eastern Asia is not identical to the European winter mushroom *F. velutipes* and should be treated as a separate species, namely *F. filiformis,* which includes all cultivated enokitake strains in East Asia and those from South Korea and Japan with genome sequences [2]. Thus, we apply the name “*F. filiformis*” instead of the Asian *F. velutipes* in our study*.*

*F. filiformis* is one of the most important and popular edible mushrooms available commercially in China. It is widely cultivated and consumed in Asian countries due to its high nutritional value and desirable taste. It has been reported that China is currently the largest producer of *F. filiformis*, with an annual production of 2.4 million tons [3]. *F. filiformis* also possesses tremendous pharmaceutical value, and many bioactive constituents have been identified, such as polysaccharides [4–6], flavonoids [7], sesquiterpenes, glycosides, proteins, and phenols [8–10]. These compounds have been shown to exhibit antitumour, anticancer, anti-atherosclerotic thrombosis inhibition, anti-aging and antioxidant effects [11, 12]. In addition, as a typical white-rot fungus, *F. filiformis* can effectively degrade lignin and produce alcohol dehydrogenase, and thus exhibiting potential for application in bioethanol production [13].

In recent decades, research has mainly focused on the phylogenetic taxonomy [1, 14], genetic diversity [15, 16], nutritional and chemical constituents [17–19], pharmacological bioactivity [20, 21] and artificial cultivation of *Flammulina* spp. [22–24]. Most studies have shown that *F. filiformis* possesses relatively high carbohydrate, protein and amino acids contents and low fat or lipid contents; thus, it generally was recognized as a low energy delicacy [25]. In addition, bioactive polysaccharides (e.g., glucans and heteropolysaccharides), immunomodulatory proteins (e.g., FIP-fve) and multiple bioactive sesquiterpenes were also isolated and identified from the fermentation broth, mycelia and fruiting bodies of *F. filiformis* [26]*.* Tang et al. [12] reviewed the compounds derived from the *F. filiformis* and their diverse biological activities. Increasing studies on the chemical compounds and biological activities of this mushroom have supported that *F. filiformis* should be exploited as a valuable resource for the development of functional foods, nutraceuticals and even pharmaceutical drugs [27].

The development of genomic and transcriptomic sequencing technologies has provided the powerful tools to understand the biology of edible mushrooms, including the effective utilization of cultivation substrates (lignocellulose) [28, 29], the mechanism of fruiting body formation and development and adaption to adverse environments, such as high temperature environments or cold-stress conditions [30–32]. For example, genome sequencing of the cultivars of *F. filiformis* from Korea and Japan revealed their high capacity for lignocellulose degradation [28, 33]. Transcriptomic and proteomic analyses of *F. filiformis* revealed key genes associated with cold- and light-stress fruiting body morphogenesis [34]. These studies provided important information for the breeding and commercial cultivation of *F. filiformis*.

Recent advances in genome sequencing have revealed that a large number of putative biosynthetic gene clusters (BGCs) are hidden in fungal genomes [35, 36]. Genome mining efforts have also allowed us to understand the silencing or activation of biosynthetic pathways in microbes with the development of bioinformatics software, such as antiSMASH, SMURF and PRISM [37]. For instances, the genome-wide investigation of 66 cosmopolitan strains of *Aspergillus fumigatus* revealed 5 general types of variation in secondary metabolic gene clusters [38]. The identification of the tricyclic diterpene antibiotic pleuromutilin gene clusters on the genome-scale increased antibiotic production in *Clitopilus passeckerianus* [39]; the prediction of gene clusters involved in the biosynthesis of terperoid/ polyketide synthase (PKS) in the medicinal fungus *Hericium erinaceus* by genome and transcriptome sequencing discovered a new family of diterpene cyclases in fungi [40, 41], and the identification of the candidate cytochromes P450 gene cluster possibly related to triterpenoid biosynthesis in the medicinal mushroom *Ganoderma lucidum* by genome sequencing improved the production of effective medicinal compounds [42, 43].

However, as a popular edible mushroom that has a wide spectrum of interesting biological activities, little is known about the synthesis and regulation of bioactive secondary metabolites of *F. filiformis*. In previous experiments, we collected the wild strain of *F. filiformis* Liu355 from Longling, Yunnan and demonstrated that it could tolerate relatively high temperatures during fruiting body formation (at 18 °C–22 °C) in the laboratory and that its temperature tolerance was superior to that of the commercial strains of *F. filiformis* that usually produce fruiting bodies at low temperatures ≤15 °C [16]. Thus, the wild strain is a potential and an important material for future breeding or engineering of new *F. filiformis* strains because increasing the temperature tolerance can save a substantial amount of energy. Most interestingly, the chemical composition of the wild strain was different from that of other commercially cultivated strains of *F. filiformis,* harboring more unique chemical compounds. A total of 13 new sesquiterpenes with noreudesmane, spiroaxane, cadinane, and cuparane skeletons were isolated and identified from the wild strain Liu355 [9]. Fungi in Basidiomycota can produce diverse bioactive sesquiterpenes but the knowledge about sesquiterpene synthases (STSs) in these fungi are unclear. The identification of sesquiterpene synthases from *Coprinus cinereus* and *Omphalotus olearius* provided useful guidance for the subsequent development of in silico approaches for the directed discovery of new sesquiterpene synthases and their associated biosynthetic genes [44].

Thus, the aims of our study are to explore the genetic features of this interesting wild strain of *F. filiformis* on a genomic scale, to predict the genes or gene clusters involved in the biosynthesis of polysaccharide or secondary metabolites and to profile the expression differences in these candidate genes during the development of *F. filiformis*. In addition, the genes related to its high-temperature-tolerance are also discussed. This research will facilitate our understanding of the biology of the wild strain, provide useful datasets for molecular breeding, improving compound production and improve the production of novel compounds by heterologous pathway and metabolic engineering in the future.

## Results

### General features of the *F. filiformis* genome

Prior to our study, three genomes classified as *F. filiformis* were available in public databases: the relatively complete genome of strain KACC42780 from Korea, a draft genome of TR19 from Japan and L11 from China (previously named as Asian *F.velutipes*). In this study, we sequenced the genome of a wild strain of *F. filiformis* by small fragment library construction and performed a comparative genomic analysis of secondary metabolite gene clusters. The assembled genome of wild *F. filiformis* was 35.01 Mbp with approximately 118-fold genome coverage. A total of 10,396 gene models were predicted, with an average sequence length of 1445 bp. The genome size and the number of predicted protein-encoding genes were very similar to the public published genome of *F. filiformis* (Table 1). Functional annotation of the predicted genes showed that more than half the predicted genes were annotated in the NCBI Non-Redundant Protein Sequence Database (NR) (6383 genes) and 5794, 2582, 1972 and 837 genes were annotated in the databases Gene ontology (GO), Kyoto Encyclopedia of Genes and Genomes (KEGG), Clusters of Orthologous Groups (COG) and SwissProt, respectively. In addition, the wild *F. filiformis* genome contained 107 cytochrome P450 family genes and 674 genes encoding secretory proteins.

**Table 1:** Genomic features of four strains of *Flammulina filiformis* (=Asian *F. velutipes*)

Strain voucher	Liu355	L11	TR19	KACC42780
Accession number	PRJNA531555	PRJNA191865	PRJNA191921	PRJDB4587
strain original	Wild, Yunnan, China	Clutivar, Fujian, China	Cultivar, Japan	Cultivar, Korea
Genome size (Mb)	35.01	34.33	34.79	35.64
Genome Coverage	118×		132×	37.2×
No. of Scaffolds	2040	1858	5130	11
No. of Contigs	2060	28 590	6 405	500
Genes number	10 396	11 526	10 096	11 038
Gene total length (bp)	15 027 318(42.92%)	17 020 883 (49.58%)	14 905 273 (42.84%)	15 924 075 (44.68%)
Gene average length	1 445	1 477	1 476	1 443
G+C content(%)	52.31	52.46	52.35	52.31
P450	107	144	-	-
CAZy	270	315	-	392
Secretory Protein	674	-	-	-
Transposon pre number	204	215	245	285

**Fig. 1:**
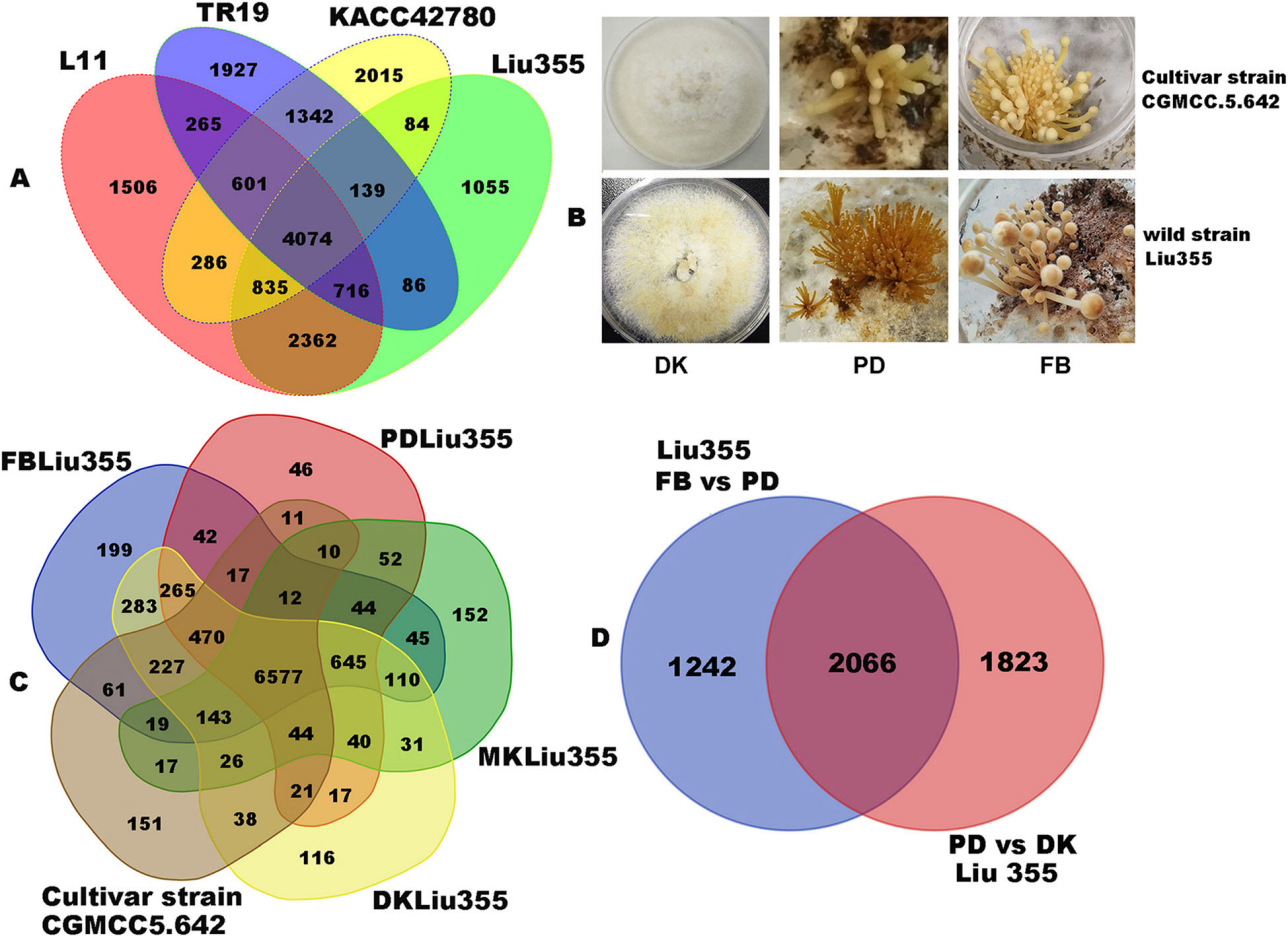
Samples information and venn diagram showing the numbers of orthologue genes or differentially expressed genes. **a** The numbers of orthologue genes between four strains of *F. filiformis*. L11(China) in red, TR19 (Japan) in purple, KACC42780 (Korea) in yellow and Liu355 (China) in green. **b** The samples of wild and cultivar strains of *F. filiformis*. Up-line: cultivar strain; down-line: wild strain, from left to right: Dikaryotic mycelium (DK); Primordium (PD) and Fruiting bodies (FB). **c** The numbers of differentially expressed genes (DEGs) in various comparative groups of *F. filiformis*. Fruiting body of the wild strain (FB) in blue, Primordium of the wild strain in red, Monokaryotic mycelium of the wild strain (MK) in green, Dikaryotic mycelium of the wild strain (DK) in yellow and Dikaryotic mycelium of the cultivar strain of *F. filiformis* in brown. **d** Venn diagram showing the numbers of DEGs at adjacent development stage of *F. filiformis*. Blue color represented the number of DEGs of fruiting body (FB) versus primordium (PD) and red color represented primordium (PD) versus dikaryotic mycelium (DK) of the wild *F. filiformis* strain. Abbreviation: MK: monokaryontic mycelium; DK: dikaryontic mycelium; PD: primordium; FB: fruiting body

Comparative genome analysis of four strains of *F. filiformis* showed that the *F. filiformis* can be described by a pan-genome consisting of a core genome (4074 genes) shared by four strains (on average 23.5% of each genome) and a dispensable genome (13,219 genes) (Fig. 1a). A total of 3104 orthologous genes were annotated in the KEGG database, 2722 genes were annotated in the GO database and 1055 genes were specific to the wild strain Liu355.

### Functional characteristics of the predicted genes of *F. filiformis*

Functional annotation in KEGG database showed that the abundance of the predicted genes of *F. filiformis* involved in translation (253 genes) was the highest, followed by carbohydrate metabolism with 243 genes. Twenty-one genes were involved in terpenoid and polyketide biosynthesis (Additional file 1: Fig. S1).

### Transcriptomic analysis and gene expression

We studied the gene expression differences across different developmental stages, namely the monokaryotic (MK), dikaryotic mycelium (DK), primordium (PD) and fruiting body (FB) stage of the wild strain *F. filiformis* Liu355. Moreover, the DK of the cultivar strain of *F. filiformis* (CGMCC 5.642) was also subjected to transcriptome sequencing (Fig. 1b). Three biological replicates were designed for each sample. The average clean data for each sample was 8.07–9.32 G. We mapped the clean reads to the genome of *F. filiformis* Liu 355 using HISAT software and obtained a relatively high total mapping rate (92.63%). In addition, the expression variation between samples was the smallest between the DK and FB stages (the average value of R^2^ = 0.85) and was the greatest between the wild strain’s MK and cultivar DK stages of the wild *F. filiformis* strain (Additional file 2: Fig. S2).

Among the 10,396 gene models of *F. filiformis*, 9931 gene models were expressed (FPKM > 5) across the four different tissues (MK, DK, PD and FB) of the wild strain and the dikaryotic mycelium of a cultivar strain of *F. filiformis*. A total of 6577 genes were commonly expressed in all tissues. One hundred fifty-one genes were specifically expressed in the cultivar strain, and 199, 152, 116, 46 genes were specifically expressed in FB, MK, DK and PD of the wild strain of *F. filiformis*, respectively (Fig. 1c). The tissue-specific and high expression transcripts in *F. filiformis* Liu355 are listed in Additional file 3: Table S1. Two genes encoding ornithine decarboxylase (involved in polyamine synthesis) were highly expressed in the mycelium of the cultivar strain (Nove l01369, Nove l01744), and the genes encoding oxidoreductase also had the highest expression level (gene 830, FPKM > 1000). The genes encoding agroclavine dehydrogenase, acetylxylan esxterase, β-glucan synthesis-associated protein and arabinogalactan endo-1,4-β-galactosidase protein were significantly highly expressed in the FB of the wild *F. filiformis* strain, with a more than 20–100-fold change compared to their expression in the mycelium. Agroclavine dehydrogenase is involved in the biosynthesis of the fungal ergot alkaloid ergovaline [45] and-β-glucan synthesis-associated protein is likely linked to the biosynthesis of fungal cell wall polysaccharides. The high expression of these genes indicates that they probably play an important role in fruiting body development and compound enrichment.

A total of 5131 genes (51.67%) were up or downregulated in at least one stage of transition, such as from mycelium to primordium (PD vs DK, 3889 genes) and from primordium to fruiting body (FB vs PD, 3308 genes) (Fig. 1d). During primordial formation, 1780 genes are upregulated, and most of the genes were annotated as oxidoreductase activity (GO:0016491), hydrolase activity (GO:0004553) and carbohydrate metabolism (GO:0005975). The downregulated genes were mainly enriched in transmembrane transport (GO:0055085). During fruiting body development, genes related to the fungal-type cell wall (GO:0009277) and the structural constituent of the cell wall (GO:0005199) were upregulated, reflecting the dramatic changes in cell wall structure during the developmental process. In addition, GO term enrichment of differentially expressed genes (DEGs) between the wild strain Liu355 and cultivar strain CGMCC 5.642 showed that most genes displayed a similar expression profile, but peptide biosynthetic and metabolic process (GO:0006518; GO:0043043), amide biosynthetic process (GO: 0043604) and ribonucleoprotein complex (GO: 1901566) were upregulated in the cultivar strain of CGMCC 5.642.

**Fig. 2:**
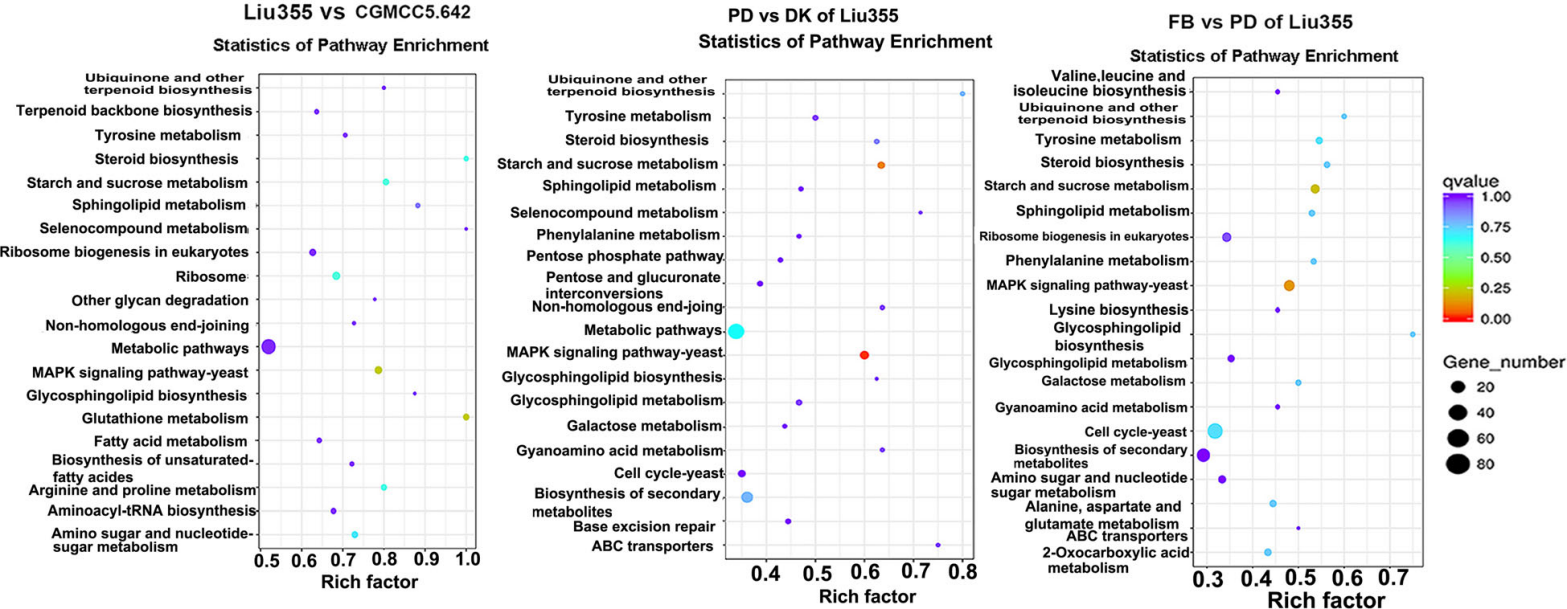
KEGG pathway enrichment analysis of differentially expressed genes (DEGs) during *F. filiformis* development. Left columns: pathway enrichment at mycelium stage of wild strain Liu355 compared to cultivar strain CGMCC 5.642; Middle columns: pathway enrichment at primordium stage compared to mycelium stage of wild strain Liu355; Right columns: pathway enrichment at fruiting body stage compared to primordium stage. Abbreviation: MK: monokaryontic mycelium; DK: dikaryontic mycelium; PD: primordium; FB: fruiting body

KEGG enrichment analysis showed that DEGs involved in glutathione metabolism were significantly enriched in DK of the wild strain Liu 355 compared to the cultivar strain (Fig. 2). Thirty-three DEGs, including genes encoding glutathione S-transferase, ribonucleoside-diphosphate reductase, 6-phosphogluconate dehydrogenase, cytosolic non-specific dipeptidase, gamma-glutamyltranspeptidase, and glutathione peroxidase, participated in this pathway. In addition, during the primordial and fruiting body development stages, the MAPK signaling pathway (45 DEGs) and starch and sucrose metabolism pathway (26 DEGs) were significantly enriched. Tyrosine metabolism, biosynthesis of secondary metabolites and glycosphingolipid biosynthesis were also significantly enriched in the fruiting body formation stage.

### Genes involved in polysaccharide biosynthesis in *F. filiformis*

We identified a total of 80 genes related to polysaccharide (PS) biosynthesis involved in glycolysis and gluconeogenesis in the KEGG pathway analysis (KEGG map 00010) [46] at the genomic level, including glucose-6-phosphate isomerase (GPI), fructose-1,6-biphosphatase (FBP), and mannose-6-phosphate isomerase (MPI). Genes encoding Zinc-type alcohol dehydrogenase were upregulated in both the mycelium of the wild strain compared to the cultivar strain and in the fruiting body compared to the mycelium of the wild of *F. filiformis* strain (Additional file 4: Fig. S3 and Additional file 5: Table S2). The genes encoding glycerol 2-dehydrogenase (gene9557, gene2028), 7-bisphosphatase (gene 2929), alcohol dehydrogenase (gene7891-D2, gene 9773-D2) and aryl-alcohol dehydrogenase (gene 4871, gene 612) were upregulated in mycelium of the wild strain. The expression level of the gene encoding mannose-1-phosphate guanylyltransferase (GDP) (gene 11,132-D3) was the highest in the mycelium of the wild strain, with a more than 200-fold change compared to that in the mycelium of the cultivar strain. The genes encoding glycerol 2-dehydrogenase (gene 894) and sugar phosphatase (gene 11,052-D2) were upregulated in the fruiting body stage of the wild strain.

To identify PS related genes, several predicted metabolic enzymes related to PS biosynthesis in *G. lucidum* [47] were also blasted by homology searches in the *F. filiformis* genome. We identified 21 putative essential enzymes involved in PS biosynthesis in *F. filiformis*, including GPI, MPI, UDP-glucose dehydrogenases (UGD), UDP-glucose pyrophosphorylase (UGP), hexokinase, galactokinase and transketolase (Table 2). Among them, genes encoding UGP, UGD and fructose-bisphosphate aldolase (FDA) had relatively high transcript levels in all samples analyzed (FPKM > 100).

**Table 2:** Putative enzymes involved in PS biosynthsis of and their gene expression in *F.filiformis*

EC No.	Gene ID	gene length	Enzyme name	FPKM mean					
				E-value	FB Liu355	PD Liu355	MK Liu355 FPKM	DK Liu355	Cultivar 5.642
5.3.1.9	gene3100	2559	Glucose-6-phosphate isomerase	**0**	66.43	69.62	104.75	95.85	80.55
2.7.1.1	gene8329	1551	Hexokinase	1E-124	71.13	75.91	100.65	90.18	72.05
2.7.1.1	gene6893	1515	Hexokinase	7E-81	54.55	133.98	63.93	124.08	132.22
5.3.1.8	gene3253	1215	Mannose-6-phosphate isomerase	1E-99	50.54	30.08	48.00	49.57	64.08
4.2.1.47	gene2044	1131	GDP-D-mannose dehydratase		77.37	69.54	139.04	106.22	122.26
2.7.7.9	gene3603	2301	UDP-glucose pyrophosphorylase	0	237.51	235.28	371.96	198.44	229.93
2.7.7.9	gene3631	4578	UDP-glucose pyrophosphorylase	4E-135	10.34	3.80	29.95	6.22	8.14
5.1.3.2	gene6737	1158	UDP-glucose 4-epimerase	3E-63	63.69	77.37	53.52	47.21	74.03
1.1.1.22	gene10364	1458	UDP-glucose dehydrogenase	9E-84	106.55	121.62	199.55	108.85	150.53
4.1.1.35	gene6505	1350	UDP-glucuronic acid decarboxylase	0	182.47	118.70	186.57	117.16	252.40
2.7.1.6	gene2127	1581	Galactokinase	3E-89	29.62	26.75	27.22	37.11	45.66
2.7.7.12	gene3782	1128	Galactose-1-phosphate uridyltransferase	6E-103	5.64	22.78	7.52	8.43	11.61
1.1.1.9	gene9850	864	D-xylose reductase	1E-106	211.66	169.46	83.98	150.70	180.47
1.1.1.14	gene10388	1218	Zinc-dependent alcohol dehydrogenase	4E-46	109.79	89.73	115.09	88.14	127.83
4.1.2.13	gene7057	1074	Fructose-bisphosphate aldolase	2E-160	359.67	334.01	354.86	298.99	304.30
3.1.3.11	gene9805	2235	Fructose-1,6-bisphosphatase	4E-117	73.51	112.62	64.35	124.76	93.38
2.7.1.17	gene52	1653	D-xylulose kinase	9E-126	12.52	15.67	2.25	8.38	5.15
2.2.1.1	gene5296	2049	Transketolase	0	234.88	171.98	187.99	189.28	138.91
2.2.1.1	gene10236	2109	Transketolase	6E-180	9.77	13.12	2.46	18.96	24.79
2.2.1.1	gene9220	2172	Transketolase	3E-172	3.54	3.86	4.42	4.41	0.41
2.7.1.11	gene4194	3438	6-phosphofructokinase	0	68.27	59.02	87.68	71.13	74.46

FPKM value is mean of three biological replicates.

*Abbreviations*: *MK* monokaryotic mycelium, *DK* dikaryotic mycelium, *FB* fruiting body, *PD* primordium

### Predicted bioactive secondary metabolite gene clusters of *F. filiformis*

**Fig. 3:**
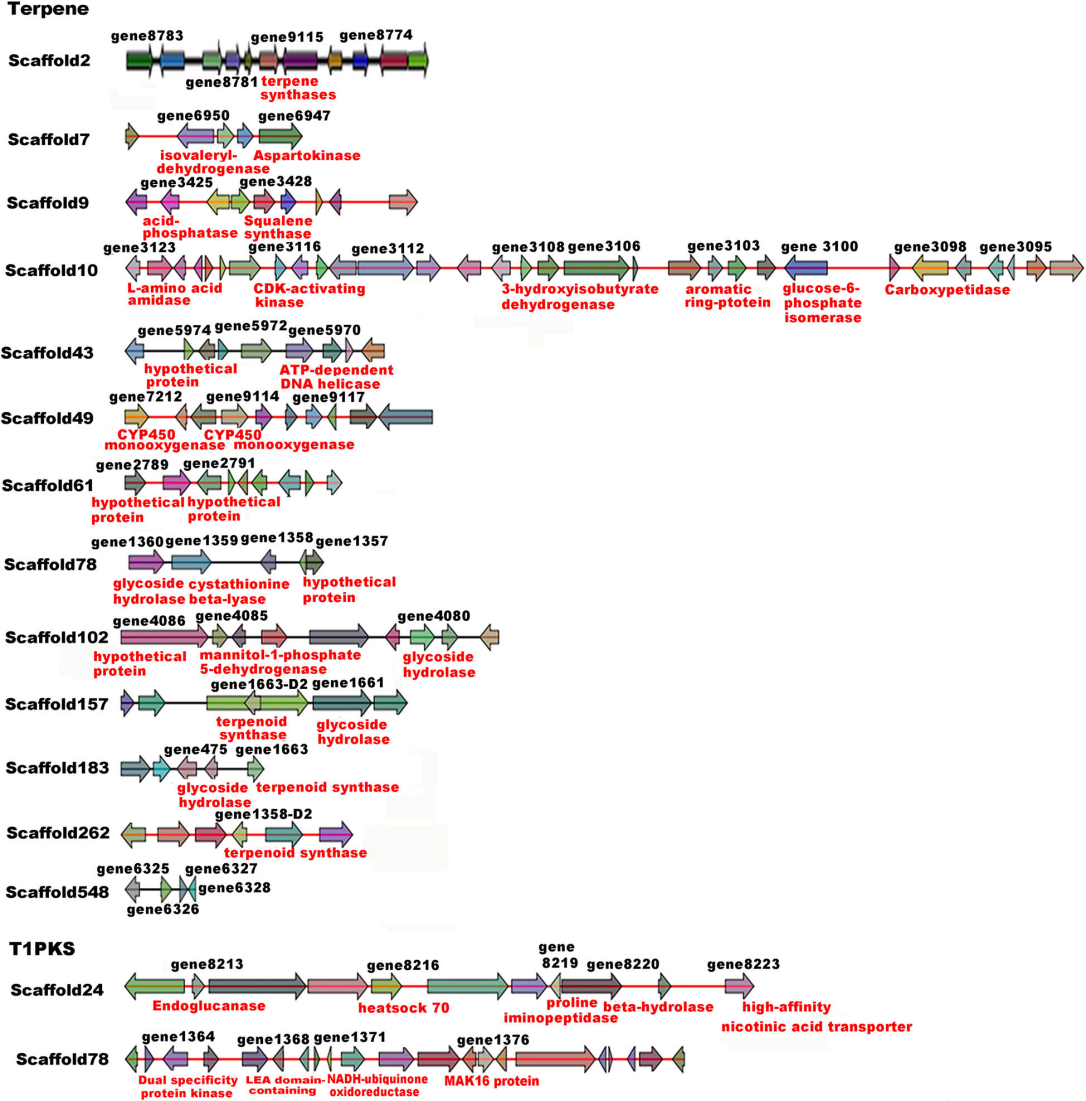
Identification of the 13 putative gene clusters for terpene and two polyketides gene clusters (PKS) in *F. filiformis* genome by antiSMASH software. Genes with SwissProt functional annotation were marked in red color

In total, 13 gene clusters related to terpenoid biosynthesis and two gene clusters for polyketide biosynthesis were predicted in the wild strain of *F. filiformis* (Fig. 3 and Additional file 6: Table S3). The numbers of gene clusters involved in terpene, PKS and NRPS biosynthesis were different in the wild strain Liu355 compared with the other three cultivar strains (KACC42780, TR19 and L11 with genome sequencing) and the gene number related to terpene synthesis was higher in the wild strain Liu355 (119 genes) than in the cultivar strain L11 (81 genes) (Table 3). We performed sequences’ similarity comparison of genes involved in predicted terpene and type I PKS gene clusters among different strains of *F. filiformis* using blastall v2.2.26 software and the result was provided in Additional file 7: Table S4. This result showed that a high similarity of genes sequence and gene cluster existence between different strains of *F. filiformis* but several individual genes specific occurred only in wild strain Liu355 compared to cultivar L11,TR19 and KACC42780 (e.g. gene cluster in scaffold 548).

**Table 3:** Putative genes and gene clusters related to secondary metabolitic biosynthesis for the *F. filiformis*

Strains	Liu 355 (wild strain)		L11(cultivar)		KACC42780(Korea)		TR19(Japan)	
Clusters	Clusters_number	gene_ number	Clusters_number	gene_ number	Clusters_number	gene_ number	Clusters_number	gene_ number
NRPS	3	22	2	20	2	59	2	17
Terpene	13	119	10	81	17	139	15	115
typeI_PKS	2	32	1	18	2	34	2	30
cf_putative	6	71	3	53	52	1286	39	696

### Putative genes for terpenoid biosynthesis in *F. filiformis*

A total of 119 genes of 13 terpenoid clusters were divided into 10 clades according to their expression levels in different developmental stages of the wild strain or cultivar strains (Additional file 8: Fig. S4). Most genes in clade II, including encoding 4,5-DOPA dioxygenase extradiol-like protein (gene3103) and squalene synthase (gene3428), which is involved in the biosynthesis of squalene, a precursor of terpenoid compounds, were upregulated in the primordium compared to the mycelium of wild strain Liu355. The genes in clades VIII-X, including key enzymes involved in terpenoid biosynthesis, such as protoilludene synthase, candidate peroxisomal acyl-coenzyme A oxidase, L-amino acid amidase and cytochorme P450, were significantly differentially expressed in the fruiting body compared with the mycelium of the wild strain Liu355 and in the mycelium of the wild strain Liu355 compared to the cultivar strain CGMCC5.642. For example, the putative terpene synthase (gene 9115) was highly expressed and significantly upregulated in the mycelium of the wild strain (1.4-fold change) compared to the mycelium of the cultivar strain (CGMCC 5.642) and in the fruiting bodies compared with the primordium in the wild strain Liu355 (19.4-fold change). Two putative cytochrome P450 genes (gene 9114 and gene 7212) were also significantly upregulated in the dikaryotic mycelium compared to the monokaryotic mycelium of the wild strain (7.61- and 7.50- fold change, respectively) and the cultivar strain mycelium (2.1- and 1.7- fold change, respectively). This result likely explained the greater diversity of bioactive compounds in the wild strain than in the cultivar strain in a previous study.

### Putative genes for sesquiterpenoid biosynthesis in *F. filiformis*

We performed a genome-scale homologous search with sesquiterpene synthases of *O. olearius, C. cinereus* and *H. erinaceus* based on the genomic data of the wild strain Liu355. Twelve homologous sequences with considerable similarity (e-value < 10^− 5^) to the known biochemically characterized sesquiterpene synthases (STS) were identified in the genome of the wild strain *F. filiformis* Liu355. Twelve STS genes of the wild *F. filiformis* strain included five genes encoding delta (6)-protoilludene synthase (gene1663-D2, gene9115, gene2784 and gene9115-D2, gene6325-D2), two genes encoding trichodiene synthase (gene1140, gene2254), two genes encoding alpha-muurolene synthase (gene1358-D2, gene1358), and one gene encoding a hypothetical protein (gene3100). The phylogenetic analysis showed that these genes grouped into four clades (Additional file 9: Fig.S5). Five genes from the wild strain Liu355 (gene1140, gene10498-D2, gene4450, gene2785, gene2254) and two genes from the cultivar strain L11 (Fla10, Fla11) of *F. filiformis* were clustered together with the cuprenene synthases Cop6 and Omp 8-Omp10 in the clade 1. These genes are likely responsible for the 1,6- or 1,7-cyclization of 3R-nerolidyl diphosphate (NPP) (Cop6) or involved in the biosynthesis of α- and β-barbatene (Omp9), compounds known to be produced by fungi and plants and carotane sesquiterpenes (Omp10). Gene 1358-D2 (from the wild strain) and Fla6 (from the cultivar strain) in clade 2 grouped with Omp1 and Omp2 and were speculated to catalyse a 1,10-cyclization of E, E-farnesyl diphosphate (FPP) to yield cadinane, a precursor of sesquiterpenoids with noreudesmane, spiroaxane, cadinane and seco-cuparane. The expression of gene1358-D2 and gene1358 in mycelium of the wild *F. filiformis* strain is significantly upregulated compared to cultivar strain based on our transcriptomic data (1.3- and 2.7- fold change, respectively) (Table 4). The genes from the wild strain (gene6325-D2, gene1663-D2, gene9115-D2 and gene9115) and from the cultivar strain of *F. filiformis* (Fla2, Fla4, Fla7 and Fla12) that clustered with Omp6 and Omp7 in clade 3 may be capable of catalyzing a 1,11-cyclization of (E, E)-FPP leading to major groups of bioactive sesquiterpenes in Basidiomycota [44]. Gene1358 and Fla9 of clade 4 clustered with Cop4, Omp4, Omp5a and Omp5b and may synthesize major compounds that require the 1, 10-cyclization of (3R)-nerolidyl diphosphate (NPP).

**Table 4:** Expression level of 12 genes encoding enzymes involved into sesquiterpenoid biosynthesis in *F. filiformis*

Gene ID	chromosome	length	wild_FB_FPKM	wild_primordia_FPKM	wild_MK_FPKM	wild_DK_FPKM	clutivar_DK_FPKM	log2ratio wild_DK/cultivar DK)	log2ratio wild_DK/MK	log2ratio wild FB/DK	log2ratio wild_FB/PD	log2 ratio wild PD/DK	Description
gene2785	Scaffold121	909	0.24	0.15	0.1	0.25	0.16	0.39	0.97	-0.02	0.2	-0.21	Trichodiene synthase
gene 1358	Scaffold78	1005	4.52	32.1	2.15	5.16	1.57	1.47	0.82	-0.15	-3.21	**3.06**	Linoleate 10R-lipoxygenase
gene1358-D2	Scaffold262	1005	45.38	48.94	124.54	82.53	38.31	0.86	-1.03	-0.82	-0.50	-0.32	Alpha-muurolene synthase
gene3100	Scaffold10	2559	66.43	69.62	104.75	95.85	80.55	0.01	-0.56	-0.49	-0.49	0.00	Hypothetic protein
gene1663-D2	Scaffold157	1035	1.87	0.17	1.34	0.27	3.49	-3.90	-2.72	2.89	3.10	-0.20	Delta(6)-protoilludene synthase
gene9115	Scaffold2	1038	111.29	4.54	2.69	21.74	10.70	0.77	**2.59**	**2.37**	**4.17**	-1.80	Delta(6)-protoilludene synthase
gene1140	Scaffold110	891	5.16	4.34	2.22	5.34	4.36	0.07	**0.84**	-0.03	-0.21	0.18	Trichodiene synthase
gene4450	Scaffold150	936	101.68	38.30	6.24	29.57	22.48	0.15	**1.81**	1.76	0.92	**0.83**	-
gene2254	Scaffold201	996	0.82	0.26	0.09	0.21	171.85	-9.93	0.71	2.04	1.25	0.79	Trichodiene synthase
gene10498-D2	Scaffold62	864	0.14	0.06	0.14	0.02	0.01	0.71	-3.10	2.65	0.75	1.89	-
gene9115-D2	Scaffold49	1038	87.98	3.11	3.31	23.40	16.45	0.25	**2.40**	**1.93**	**4.39**	-2.46	Delta(6)-protoilludene synthase
gene6325-D2	Scaffold61	1017	43.08	3.28	0.89	42.16	11.23	1.66	**5.14**	0.06	**3.27**	-3.21	Delta(6)-protoilludene synthase

FPKM value is the mean expression value of three biological replicates

black bont means genes significantly upregulated

*Abbreviation*: *MK* monokaryontic mycelium, *DK* Dikaryontic mycelium, *FB* fruiting body, *PD* primordium

### Putative genes for polyketide biosynthesis in *F. filiformis*

**Fig. 4:**
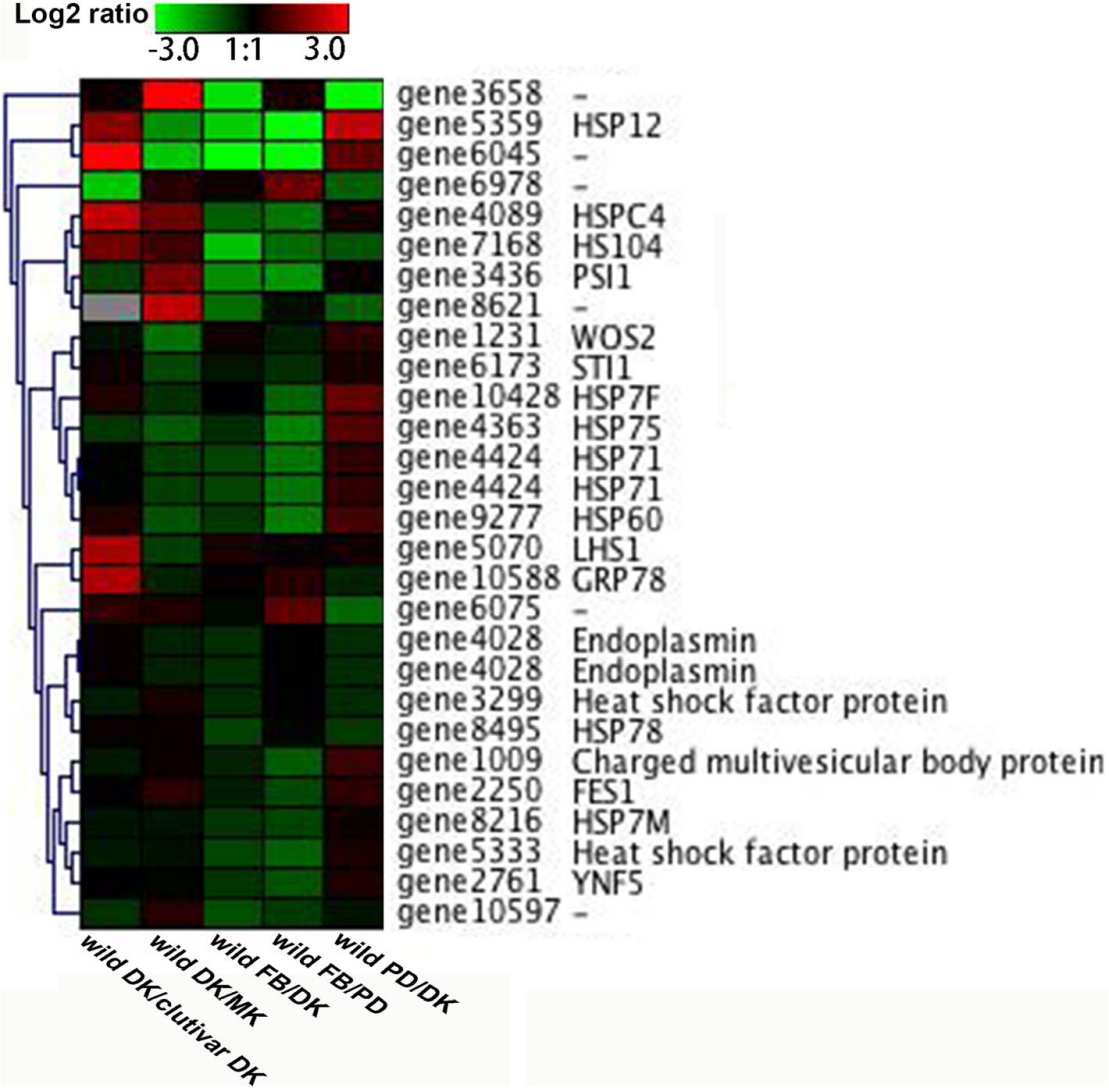
Hierarchical clustering analysis of 28 putative heat-shock protein encoding genes in *F. filiformis* genome between wild strain Liu355 and cultivar strain CGMCC5.642 and among four development stages of wild strain Liu355. Expression ratios were plotted in a heatmap on a log2 scale. The red and green colors indicate up- and down-regulation, black represents no significant expression change and grey represents missing. The abbreviation: MK, monokaryotic mycelium; DK, Dikaryotic mycelium; PD, primordium; FB, Fruiting body

The diverse structures of polyketides are biosynthesized from short-chain carboxylic acid units by polyketide synthases (PKSs), PKSs have been classified into type I, type II and type III based on their product profiles and catalytic domain architecture [48]. By gene cluster prediction using antiSMASH, we found 30 genes in two gene clusters annotated as type I PKSs, and they were mainly located in a single scaffold, 24 and 78, in the *F. filiformis* genome, respectively (Fig. 4 and Additional file 6: Table S3). The two gene clusters both included core genes encoding polyketide synthases (gene8217 and gene1373). The genes located on scaffold 78, including putative polyketide synthase (gene1373, gene1374) and benzoate 4-monooxygenase (gene 1372), were most upregulated in the wild strain mycelium compared with the cultivar strain mycelium, indicating that polypeptide compounds are probably abundant in the mycelia of this mushroom and especially in the wild strain.

### Cytochrome P450s in the *F. filiformis* genome

We identified 107 genes in the cytochrome P450 family, including nine putative trichodiene oxygenases, 31 O-methylsterigmatocystin oxidoreductases, five benzoate 4-monooxygenases, two linoleate 10R-lipoxygenases, two ent-kaurene oxidases, lanosterol 14-alpha and flavonoid hydroxylases and other candidate cytochrome P450s. Of these, 102 genes had diverse expression profiles across different tissues of *F. filiformis*. Twenty-six CYP450 genes were upregulated in the mycelium of the wild strain compared to the cultivar strain and the cytochrome P450 (gene 5820-D3) had the highest expression level, with more than a 500-fold change. Twenty-one CYP450 genes were upregulated in the fruiting body stage compared to the mycelium stage, and genes encoding benzoate 4-monooxygenases had the highest transcript level, with 15-fold change. In the primordim stage, the gene encoding docosahexaenoic acid omega-hydroxylase was the highest differentially expressed gene.

### Heat-shock proteins correspond to temperature changes in *F. filiformis*

In our study, the wild strain of Liu355 could grow fruiting bodies at 18 °C–22 °C in the laboratory. The heat-shock protein (HSP) family is known to be positively correlated with organism thermotolerance [49]. Twenty-eight genes annotated as HSPs were identified in the wild *F. filiformis* genome (Fig. 4). Among them, six genes were significantly upregulated in the wild strain Liu355 compared to the cultivar strain, including encoding protein HSP12, HSPC4, HSP104, LHS1 and GRP78, respectively. HSP12 (gene5359), HSP7F (gene10428), HSP75 (gene4363), HSP71 (gene4424) and HSP60 (gene9277) are differentially upregulated expression at the beginning of fruiting bodies formation (PD stage) compared to vegetative growth (DK stage).

## Discussion

*Flammulina filiformis* is one of most widely cultivated white rot fungi in large commercial scale in China. It was reported that the first cultivar strain of *F. filiformis* in China was domesticated from a wild strain isolated from Fujian province in 1974 [15]. To date, the genomes of three cultivars of *F. filiformis* from Japan, Korea and China were sequenced respectively. In this study, we sequenced the genome of a wild strain of *F. filiformis* with abundant sesquiterpenoid compounds and high-temperature tolerance, which collected from Yunnan Province recently. The genome size (35.01 Mbp) and the numbers of putative genes (10396) of the wild *F. filiformis* were similar to the previous public genome of the cultivar *F. filiformis* (Table 1)*.* Pan-genomic analysis indicated that only 23.5% orthologus genes were shared among the four strains of *F. filiformis* (Fig. 1a). The proportion (23.5%) of core genes in the pan-genomic analysis of *F. filiformis* was similar to that in the pan-genomic analysis of 23 *Corallococcus* spp. [50]. The number seems relative lower than actual number. A possible explain was that these genomes were different sequencing depth or different methods applied for genomic assemble and annotation.

Transcriptomic analysis showed 30 genes were specific expression in mycelium of the cultivar CGMCC5.642, including genes encoding ornithine decarboxylase (ODC), N-acetyltransferase and malate dehydrogenase; four genes without functional annotation were specific expression in dikaryotic mycelium of the wild strain *F. filiformis* (Additional file 3:Table S1).ODC was the first and rate-limiting enzyme in the synthesis of polyamines and it was also involved in methyl jasmonate-regulated postharvest quality retention in button mushrooms [51]. Specific expression of these genes in cultivar strain of *F. filiformis* was possible related to human domestic activity, while the function of the genes specifically expressed in the wild strain of *F. filiformis* will be further studied.

In addition, the genes involved in glutathione metabolism were significantly enriched in DK of the wild strain Liu 355 compared to the cultivar strain CGMCC 5.642. A study on the glutathione metabolism in the filamentous fungus *Aspergillus nidulans* indicated that glutathione itself and glutathione metabolic enzymes play crucial roles in the germination of conidiospores and markedly contribute to the general stress tolerance of the fungus [52]. The high expression of genes related to glutathione metabolism in the wild strain of *F. filiformis* implied that the strain probably had strong environmental adaptation and it would be a potential better breeding resource.

Polysaccharides (PSs) are important and bioactive components of *F. filiformis* and other edible and medicinal mushrooms [47]. GPI, FBP, UGD and UGP are known important enzymes in the biosynthetic pathway of PSs of edible mushrooms [43, 47]. In our study, genes encoding to GPI, FBP and MPI were predicted to involve in PS biosynthesis of *F. filiformis* by KEGG enrichment analysis and by homologous protein search with known enzymes involved in PS biosynthesis of medicinal mushroom of *G. lucidum*. The gene encoding mannose-1-phosphate guanylyltransferase (GDP) exhibited differential expression in mycelium of the wild strain Liu 355 compared to cultivar strain CGMCC5.642 with over 200-fold change, indicating the potential abundance of PS compounds, and the content difference of PSs between the wild and the cultivar strain of *F. filiformis* will be determined in the next study.

Besides PSs, sesquiterpene compounds are the main bioactive secondary metabolites in *Flammulina*. The chemistry investigation of six strains (one wild strain and five other cultivar strains) of *F. filiformis* in a previous report revealed that the wild strain Liu355 contained many new sesquiterpenes with various skeletons, including cuparane-type and sterpurane-type sesquiterpenes. Noreudesmane, spiroaxane, cadinane and seco-cuparane sesquiterpenoids were first identified as new compounds in the mycelium of the wild *F. filiformis* strain and 12 putative sesquiterpene synthase genes (Fla1–12) were also predicted from the genomic sequence of the cultivar strain of *F. filiformis* L11 [9]. These more sesquiterpenes from the wild strain of *F. filiformis* were mainly derived from 1,10-cyclization of FPP mechanisms. Thus, the enzymes encoded by gene1358-D2 and gene1358, clustered with known functional Omp1 and Omp2 (*O. olearius*) are probably responsible for the production of sesquiterpene with specific skeleton of the *F. filiformis*. The expression level of gene1358-D2 and gene1358 will be verified in different strains of *F. filiformis* by quantitative real-time PCR in the next step. In addition, the yield of these sesquiterpene compounds and the reason for the wild strain possessing more kinds of sesquiterpene compounds will be further explained in ongoing research.

Comparative studies of filamentous fungal species have shown that secondary metabolism gene clusters are often either highly divergent or uniquely present in one or a handful of species [38]. Investigation of genome-wide within-species variation of 66 cosmopolitan strains of a single species *Aspergillus fumigatus* revealed that genes cluster exist location polymorphisms (a cluster was found to differ in its genomic location across strains) and it affect the function of gene cluster [38]. In our study, we did a preliminary analysis of gene cluster prediction within four different strains and revealed the more than 95% genes in predicted terpene and type I PKS clusters in wild strain Liu355 also existed in other three strain. However, the location confirmation of these gene clusters and the function need to be verified by experiments in the further study. Sometimes, the number of predicted genes clusters related to secondary metabolism in fungi based on genome sequencing data was also effected on sequencing method (platform) and sequencing depth and sample size (only four strains can available in our study), therefore, more strains from different geographic regions will be collected for further analysis.

A temperature downshift (cold stimulation) is considered to be one of the most important and essential environmental factors for the fruiting initiation and fruiting body formation of *F. filiformis* [34]. In our study, six genes annotated with heat shock protein family (HSP12, HSPC4, HSP104, LHS1 and GRP78) displayed significantly differential expression in the wild strain Liu355 compared to the cultivar strain. It is known that HSP12 is part of a group of small HSPs that function as chaperone proteins and are ubiquitously involved in nascent protein folding by protecting proteins from misfolding and are partially characterized as a stress response; the expression of HSP12 protein was observed in response to cold stress [53]. The expression of HSP104 and HSP70 is regulated by the Hsf (heat-shock factor) interaction, which can be stimulated by heat stress in yeast [49]. In addition, HSP70 chaperone and two putative HSP were also found were upregulated at only primordium or young fruiting body of cultivar *F. filiformis* using the iTRAQ labeling technique [54]. Our study also predicted that genes encoding HSP12, HSP 71, HSP60 probable involved in formation and differentiation of fruiting bodies of *F. filiformis*. However, the exact molecular function of HSPs in the high-temperature tolerance of wild *F. filiformis* and its adaptive mechanisms for relatively high temperatures need further study.

## Conclusions

In our study, genome and transcriptome sequencing and the assembly and annotation of the high-temperature-tolerant wild *F. filiformis* strain were carried out, and the gene clusters associated with polysaccharides, terpenoid and polyketide biosynthesis were predicted*.* Comparative genomic analysis with three other Asian cultivar strains of *F. filiformis* revealed that the wild strain has a similar genome size and relatively more putative gene numbers related to secondary metabolite biosynthesis. Most genes related to terpenoid biosynthesis were upregulated in the primordium and fruiting body of the wild strain, while PKS genes were generally upregulated in the mycelium of the wild strain; however, the specific regulatory pathways involved such synthesis pathways remain unresolved in this study.

Six genes belonging to the HSP family, including HSP12, HSPC4, HSP104, LHS1 and GRP78, were significantly upregulated in the wild strain Liu355 compared to the cultivar strain and may be responsible for the development of fruiting bodies at relatively high temperatures in the high-temperature-tolerant wild *F. filiformis* strain. However, the expression of these genes in other strains of *F. filiformis*, especially in strains under low-temperature developmental conditions, requires verification in future studies. Our study provides an important genetic dataset for *F. filiformis* as a potential breeding material, and provides a foundation for enhancing the understanding of the biology of *F. filiformis.*

## Materials and methods

### Fungal strains and strain culture

The wild strain Liu355 used for genomic sequencing was kindly provided by Prof. H. W. Liu (State Key Laboratory of Mycology, Institute of Microbiology, Chinese Academy of Sciences) and was first isolated from the fruiting body of *F. filiformis* collected from Longling, Yunnan Province, southwestern China. The voucher specimens of the *F. filiformis* fruiting bodies were deposited in the Cryptogamic Herbarium, Kunming Institute of Botany, Chinese Academy of Sciences (HKAS85819). DNA sequencing of the internal transcribed spacer (ITS) region of *F. filiformis* (HKAS85819) was listed under GenBank accession number KP867925 [9]. The haploid monokaryotic strain *F. filiformis* Liu355 (deposited in Mycological Laboratory of the Institute of Medicinal Plant Development, Chinese Academy of Medical Sciences) was prepared by the protoplast mononuclear method and was grown on potato dextrose agar (PDA) at room temperature for 2–3 weeks in the dark. The fruiting bodies were obtained in sterile plastic bottles containing on growth substrate (cotton seed hulls, 78%; wheat bran, 20%; KH_2_PO_4_, 0.1%; MgSO_4_, 0.1%; sucrose, 1%; and ground limestone, 1%; with a moisture content of 70%) at 25 °C for 30 d, followed by cold stimulation at 18 °C and 90% humidity until primordial development occurred. Cultures were maintained at low temperature (18 °C and 75% humidity) to allow full fruiting body development [55]. In addition, the genomic data of two cultivar strains from Korea (KACC42780, Bioproject PRJNA191921) and Japan (TR19, Bioproject PRJDB4587) were available from the NCBI public database, and the genomic sequence of strain L11 (Bioproject PRJNA191865) was kindly provided by the Mycological Research Center, College of Life Sciences, Fujian Agriculture and Forestry University [56]. The cultivar dikaryotic strain (CGMCC 5.642) was obtained from the China General Microbiological Culture Collection Center (Beijing, China, http://www.cgmcc.net/) and stored in our laboratory.

### Genome and transcriptome sequencing and analysis

Total genomic DNA of *F. filiformis* was extracted from the mononuclear mycelia in PDA medium using the Omega E.Z.N.A. fungal DNA midi kit (Omega, USA) according to the manufacturer’s instructions. Total DNA was evaluated by agarose gel electrophoresis and quantified by Qubit 2.0 Fluorometer (Thermo Scientific). Library construction and sequencing was performed at the Beijing Novogene Bioinformatics Technology Co. Ltd. (China). The quality and quantity of libraries were checked using an Agilent 2100 Bioanalyzer. The *F. filiformis* strain was sequenced using 350 bp paired-end reads on an Illumina HiSeq 4000 platform via the PE150 method. The quality of sequencing results for fastq files was evaluated using software FastQC (http://www.bioinformatics.babraham.ac.uk/projects/fastqc/) and readfq.v10 (https://github.com/lh3/readfq) also was used for sequence quality control. The raw data was filtered by removing the low-quality reads, including reads with N content higher than 10%, reads whose base quality value is less than 20 with the ratio is higher than 40%, duplication (exactly the same PE reads) and PE reads containing sequencing adapters (15 bases aligned to the adapter sequence). After that, the high-quality reads were mapped to the reference genome sequence of *F. filiformis* L11 (Bioproject PRJNA191865) using BWA v0.5.9-r16 software. Functional annotation of the predicted genes was performed using BLAST against Gene Ontology (GO), Kyoto Encyclopedia of Genes and Genomes (KEGG), SwissProt and NCBI Non-Redundant Protein Sequence Database (NR) [40].

The term pan-genome was first proposed by Tettelin et al. in 2005 and it defines the entire genomic repertoire of a given phylogenetic clade and encodes for all possible lifestyles carried out by its organisms [57, 58]. Pan-genome usually comprises the core-genome (essential nucleotide sequences shared by all genomes in the cohort), dispensable genome (nucleotide sequences shared by a subset of genomes in the cohort) and strain-specific genes (nucleotide sequences existing only within a particular genome in the cohort) [59]. Pan-genome analysis in our study was carried out using the standalone CD-HIT tool to cluster orthologous proteins [60].

For transcriptomic sequencing, total RNA was extracted using the RNAeasy Plant Mini kit (Qiagen) according to the manufacturer’s protocols. Five samples were prepared; the monokaryotic mycelium (MK), dikaryotic mycelium (DK), primordium (PD) and fruiting bodies (FB) of the wild strain Liu355 and the dikaryotic mycelium of the cultivar strain CGMCC 5.462. Each sample had three biological replicates. All samples were subjected to RNA-Seq on the Illumina HiSeq2000 platform (Illumina, San Diego, CA, USA). Raw data (raw reads) of fastq format were firstly processed through FastQC (http://www.bioinformatics.babraham.ac.uk/projects/fastqc/). In this step, clean data (clean reads) were obtained by removing reads containing adapter that were added for reverse transcription and sequencing, low-quality bases (> 50% of the bases with a quality score ≤ 5), reads containing ploy-N and low quality reads that sequences containing too many unknown bases (> 5%). At the same time, Q20, Q30 and GC content the clean data were calculated. All the downstream analyses were based on the clean data with high quality. After that, the RNA-seq reads were mapped to the *F. filiformis* genome (Liu355) using TopHat v2.0.1253 [61]. HTSeq v0.6.1 software was used to count the read numbers mapped to each gene [62]. The FPKM value was used to calculate gene expression, and the upper-quartile algorithm was used to correct the gene expression. Gene differential expression analysis was performed using the DESeq R package (1.10.0) using a corrected *p*-value [63]. Genes with an adjusted *P*-value < 0.05 were considered differentially expressed. Hierarchical clustering of gene expression was conducted using Genesis 1.7.7 [64].

### KEGG enrichment analysis of differentially expressed genes (DEGs)

We used KOBAS v2.0 software to test the statistical enrichment of differentially expressed genes (DEGs) in KEGG pathways (https://www.kegg.jp/kegg/). Based on the hypergeometric distribution, we predicted the enriched pathway of DEGs with all the annotation genes. The formula is below: N represented the all gene number with pathway annotation, n is the DEGs number of N, M refers the all gene number annotated in a specific pathway and m is the DEGs number annotated in a specific pathway. The pathway was defined as significant enrichment (Padj≤0.05).

$$ p=1-\sum \limits_{i=0}^{m-1}\frac{\left(\underset{i}{M}\right)\left(\underset{n-i}{N-M}\right)}{\left(\underset{n}{N}\right)} $$

### Prediction of gene clusters involved in biosynthesis of secondary metabolites

The biosynthetic gene clusters were predicted using antiSMASH 3.0 software [65]. AntiSMASH currently offers a broad collection of tools and databases for automated genome mining and comparative genomics for a wide variety of different classes of secondary metabolites [66]. In addition, Sequence homology searches method (BlastP) was also used to identify genes related to terpenoid biosynthesis. The sesquiterpene synthases were identified based on multiple sequence alignments and phylogenetic analyses developed by the Schmidt-Dannert group [44]. It has been reported that a more divergent cytochrome P450 oxidase could be involved in secondary biosynthesis [67]. Therefore, we searched the genome of *F. filiformis* for proteins with a P450 conserved domain using the NCBI CDD tool and BLASTp [40] and also by homology BLAST in the Fungal Cytochrome P450 Database (p450.riceblast.snu.ac.kr/index.php? a = view) to obtain the annotated gene for cytochrome P450. In addition, CAZymes were identified using the Carbohydrate Active enZymes (CAZy) database [68]. We performed DIAMOND search against the CAZy pre-annotated CAZyme sequence database and combined with corresponding gene functional annotation to get the annotation result. TransposonPSI software was used as transposon gene prediction and this software uses PSI-BLAST to detect distant homology between genomic sequences and a TE library bundled with the program [69]. Secretory proteins was predicted with Signal P4.1 server (http://www.cbs.dtu.dk/services/signalP/) searching in all encoding amino acid sequence of *F. filiformis* genome*.*

## Supplementary information

**Additional file 1: Fig.S1.** A KEGG functional annotation of the predicted genes of *F. filiformis*. The highest number of genes related to metabolism process and carbohydrate metabolism except for genetic information processing.

**Additional file 2: Fig.S2.** Relationships among five transcriptomes samples of *F. filiformis*. Pairwise correlation of normalized FPKMs between RNA samples. The Pearson correlation coefficient ranges from no correlation (white) to perfect correlation (dark blue). Each sample has three biological replicates. The abbreviation: MK, monokaryotic mycelium; DK, Dikaryotic mycelium; PD, primordium; FB, Fruiting body.

**Additional file 3: Table S1.** Tissue-specific expression transcript in four different tissues of *F. filiformis. (XLSX 52 kb)*

**Additional file 4: Fig. S3.** KEGG mapping (map 00010) of glycolysis/gluconeogenesis pathway [46] identified in *F. filiformis* and the putative gene expression level on different tissue of *F. filiformis*. Red stars indicate the hits of differentially expressed genes in this map. The expression level of mapped genes (EC 5.4.2.2, EC 5.3.1.9, EC 5.3.1.1, EC 4.2.1.11, EC 1.2.4.1, EC 1.1.1.1, EC1.1.1.2) in different tissues was displayed in map. Abbreviation: F1, dikaryotic mycelium of cultivar strain CGMCC 5.642; F2, dikaryotic mycelium of wild strain Liu355; F3, fruiting body of wild strain Liu355; F4, primordium of wild strain Liu355; F5, monokaryotic mycelium of wild strain Liu355. The red and green colors indicate up-and down-regulation; black represents no significant expression change. Detail information of about the gene can be found in Additional file 5 (Note: obtained appropriate copyright permission to use the map from KEGG).

**Additional file 5: Table S2.** The expression of genes involved in glycolysis and gluconeogenesis pathway in *F. filiformis* genome.

**Additional file 6: Table S3.** Predicted biosynthetic gene clusters involved in terpene, PKS, NRPS and siderophore in *F. filiformis* using antiSMASH tool.

**Additional file 7: Table S4.** A comparative analysis of distribution of putative genes and gene clusters (terpene and type I PKS) related to secondary metabolitic biosynthesis in different strains of the *F. filiformis*. A blast search was performed using the software blastall v2.2.26 and thethreshold parameter: identity > 40% coverage > 40%. “1”: implied the gene distribution in other strain and “0” means the gene probabaly is not distribution in other strain.

**Additional file 8: Fig. S4.** Hierarchical clustering analysis of 119 putative genes related to terpenoid biosynthesis in *F. filiformis* genome. Expression ratios were plotted in a heatmap on a log2 scale. The red and green colors indicate up- and down-regulation, black represents no significant expression change and grey represents missing data. The abbreviation: MK, monokaryotic mycelium; DK, Dikaryotic mycelium; PD, primordium; FB, Fruiting body. Detail information of about the gene annotation can be found in Additional file 6.

**Additional file 9: Fig. S5.** Neighbor-Joining phylogram of putative sesquiterpene synthases (STS) of *F. filiformis* were constructed based homologous protein sequences. The number along branch represent the bootstrap value above 50%. The gene encoding sesquiterpene synthases with red dot were identified in this study. Detail information of the sequences used in phylogram can be found in reference [40, 44]. Labeled in “Cop” from the fungus *Coprinopsis cinereus*; “Omp” from fungus *Omphalotus olearius* and “Sh” from fungus *Stereum hirsutum. (JPG 439 kb)*

## Data Availability

The datasets supporting the results of this article are included with in the article and additional files. This Whole Genome Shotgun project has been deposited at DDBJ/ENA/GenBank under the accession JACYFH000000000 (https://submit.ncbi.nlm.nih.gov/subs/genome/). The original transcriptomic data have been deposited in public SRA database (accession No.PRJNA530834) (https://www.ncbi.nlm.nih.gov/sra/PRJNA530834) for tanscriptome. DNA sequencing (ITS region) of *F. filiformis* (KP867925) was listed https://www.ncbi.nlm.nih.gov/nuccore/KP867925. The public genome information of *F. filiformis* (previously known as Asian *F. velutipes*) can be available in Genbank database for PRJNA191921(https://www.ncbi.nlm.nih.gov/bioproject/PRJNA191921), PRJDB4587 (https://www.ncbi.nlm.nih.gov/bioproject/?term=PRJDB4587), PRJNA191865 (https://www.ncbi.nlm.nih.gov/bioproject/?term=PRJNA191865).

## Abbreviations

AA: Auxiliary Activitie
BGCs: Biosynthetic gene clusters
CAZymes: Carbohydrate-Active Enzymes
CBM: Carbohydrate-binding module
CE: Carbohydrate Esterases
CDD: Conserved Domain Database
COG: Clusters Orthologous Groups
CYP: Cytochrome P450 proteins
DEGs: Differentially expressed genes
DK: Dikaryotic mycelium
FB: Fruiting body
FBP: Fructose-1,6-bisphosphatase
FDA: Fructose-bisphosphate aldolase
FPKM: Fragments Per Kilobase of transcript per Million fragments mapped
FVPs: *F. velutipes* polysaccharides
GDP: Mannose-1-phosphate guanylyltransferase
GH: Glycoside-Hydrolases
GK: Glucokinase
GO: Gene Ontology
GPI: Glucose-6-phosphate isomerase
GT: Glycosyltransferases
HSP: The heat shock protein
Hsf: Heat-shock factor
KEGG: Kyoto Encyclopedia of Genes and Genomes
MAPK: Mitogen-activated protein kinase
MK: Monokaryotic
MPI: Mannose-6-phosphate isomerase
PD: Primordium
PDA: Potato dextrose agar
PGI: Phosphoglucose isomerase
PGM: Phosphoglucomutase
PKS: Polyketide synthase
PSs: Polysaccharides
UGP: UDP-glucose pyrophosphorylase
PL: Polysaccharide Lyases
STSs: Sesquiterpene synthases
ITS: Internal transcribed spacer
