# Supplementary figures and images for: Genome-wide analysis and prediction of genes involved in the biosynthesis of polysaccharides and bioactive secondary metabolites in high-temperature-tolerant wild Flammulina filiformis
Source: BMC Genomics. 2020 Oct 17;21:719. doi: 10.1186/s12864-020-07108-6 (PMC7568368; doi:10.1186/s12864-020-07108-6)

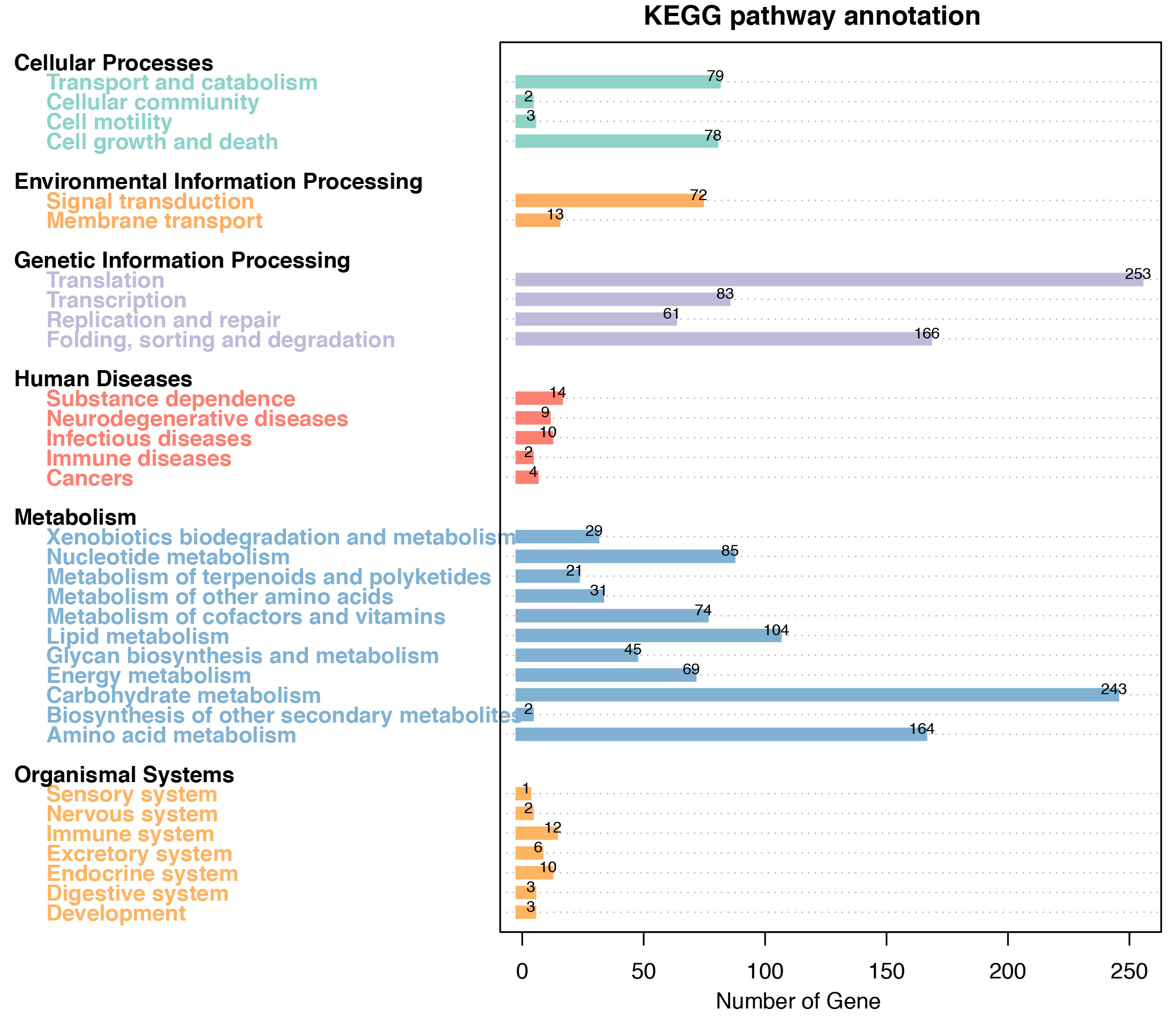

Supplement: Supplementary file 1 — Additional file 1: Fig.S1. A KEGG functional annotation of the predicted genes of F. filiformis. The highest number of genes related to metabolism process and carbohydrate metabolism except for genetic information processing. [file 12864_2020_7108_MOESM1_ESM.jpg]

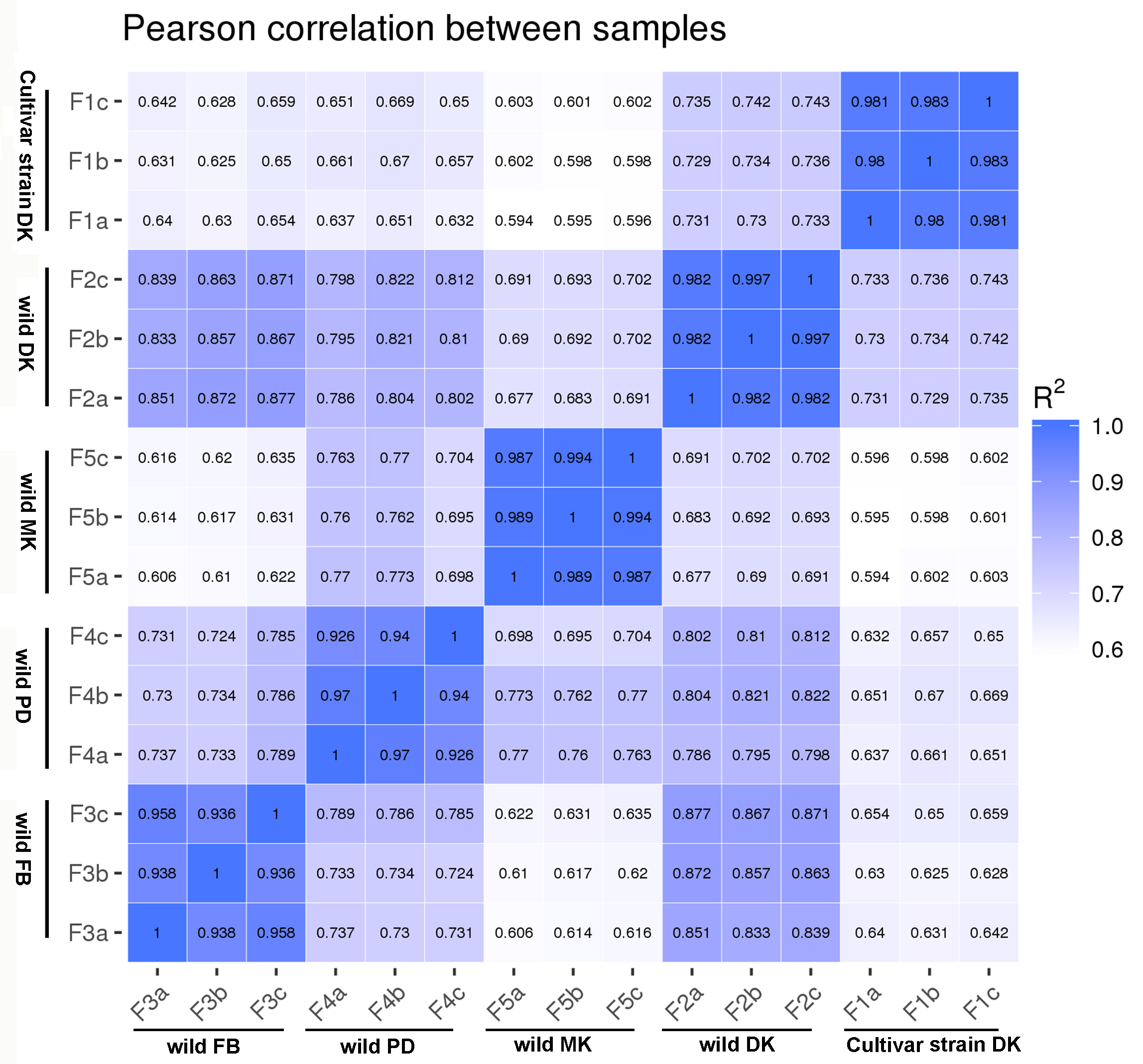

Supplement: Supplementary file 2 — Additional file 2: Fig.S2. Relationships among five transcriptomes samples of F. filiformis. Pairwise correlation of normalized FPKMs between RNA samples. The Pearson correlation coefficient ranges from no correlation (white) to perfect correlation (dark blue). Each sample has three biological replicates. The abbreviation: MK, monokaryotic mycelium; DK, Dikaryotic mycelium; PD, primordium; FB, Fruiting body. [file 12864_2020_7108_MOESM2_ESM.jpg]

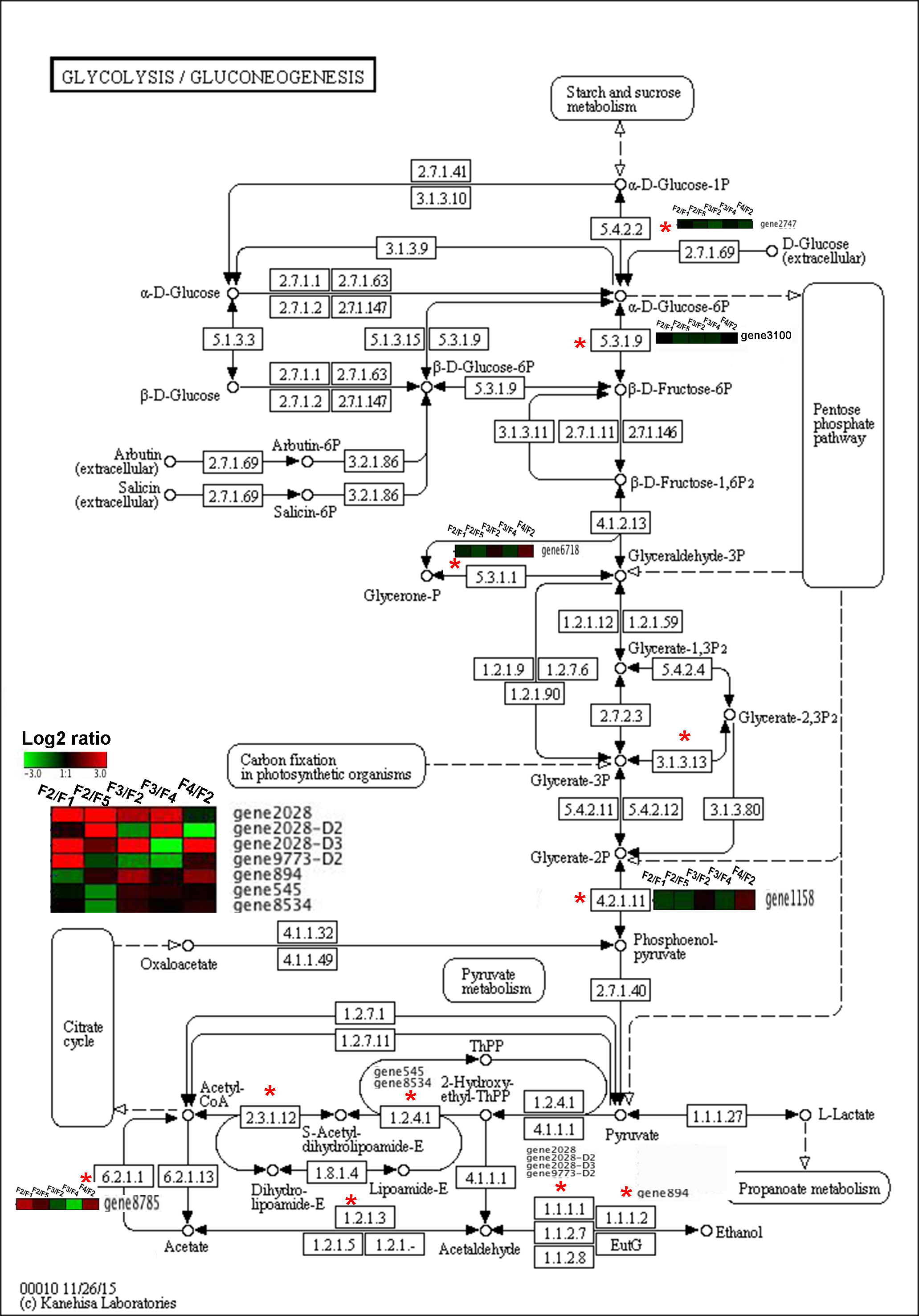

Supplement: Supplementary file 4 — Additional file 4: Fig. S3. KEGG mapping (map 00010) of glycolysis/gluconeogenesis pathway [46] identified in F. filiformis and the putative gene expression level on different tissue of F. filiformis. Red stars indicate the hits of differentially expressed genes in this map. The expression level of mapped genes (EC 5.4.2.2, EC 5.3.1.9, EC 5.3.1.1, EC 4.2.1.11, EC 1.2.4.1, EC 1.1.1.1, EC1.1.1.2) in different tissues was displayed in map. Abbreviation: F1, dikaryotic mycelium of cultivar strain CGMCC 5.642; F2, dikaryotic mycelium of wild strain Liu355; F3, fruiting body of wild strain Liu355; F4, primordium of wild strain Liu355; F5, monokaryotic mycelium of wild strain Liu355. The red and green colors indicate up-and down-regulation; black represents no significant expression change. Detail information of about the gene can be found in Additional file 5 (Note: obtained appropriate copyright permission to use the map from KEGG). [file 12864_2020_7108_MOESM4_ESM.jpg]

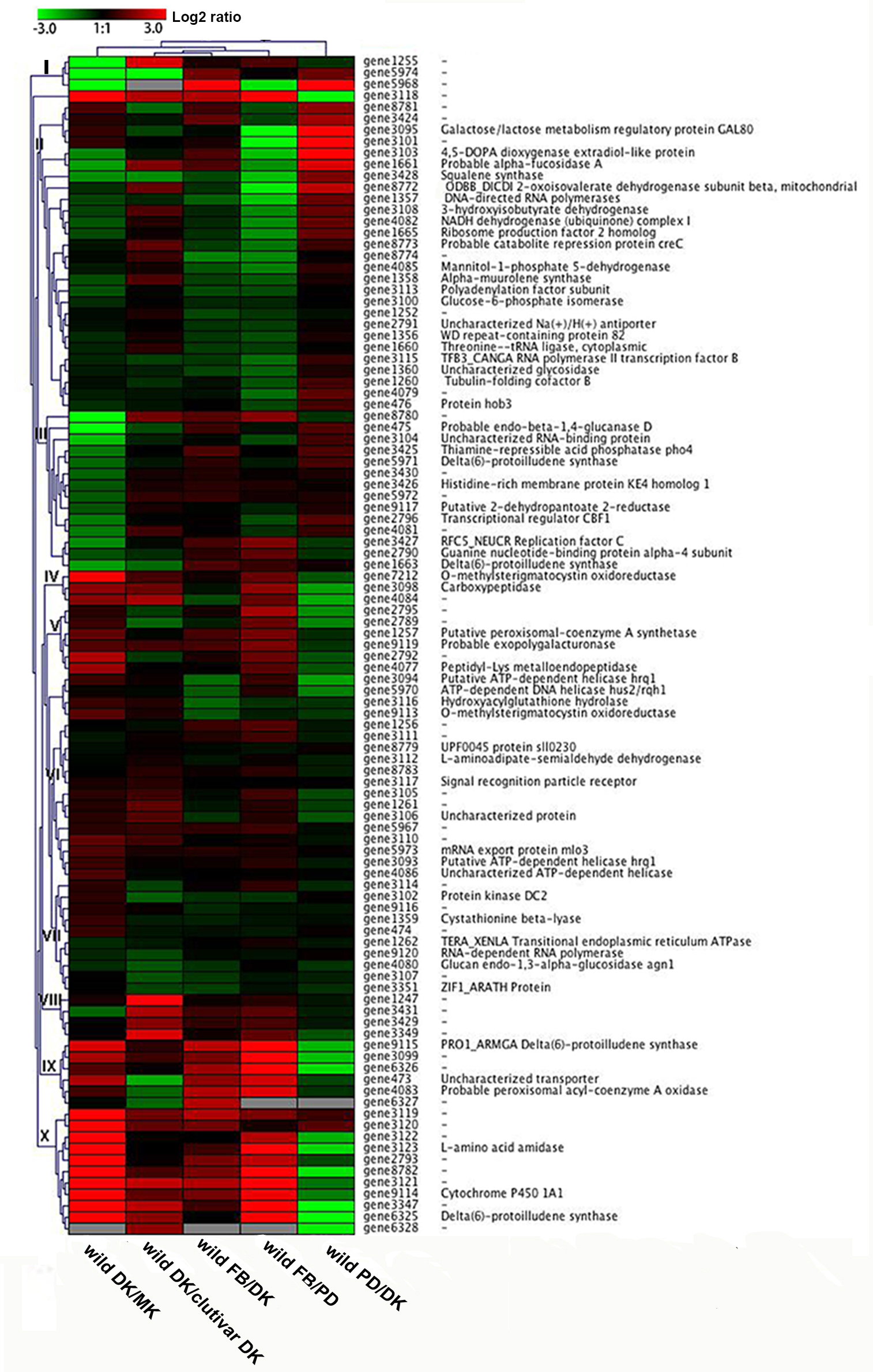

Supplement: Supplementary file 8 — Additional file 8: Fig. S4. Hierarchical clustering analysis of 119 putative genes related to terpenoid biosynthesis in F. filiformis genome. Expression ratios were plotted in a heatmap on a log2 scale. The red and green colors indicate up- and down-regulation, black represents no significant expression change and grey represents missing data. The abbreviation: MK, monokaryotic mycelium; DK, Dikaryotic mycelium; PD, primordium; FB, Fruiting body. Detail information of about the gene annotation can be found in Additional file 6. [file 12864_2020_7108_MOESM8_ESM.jpg]

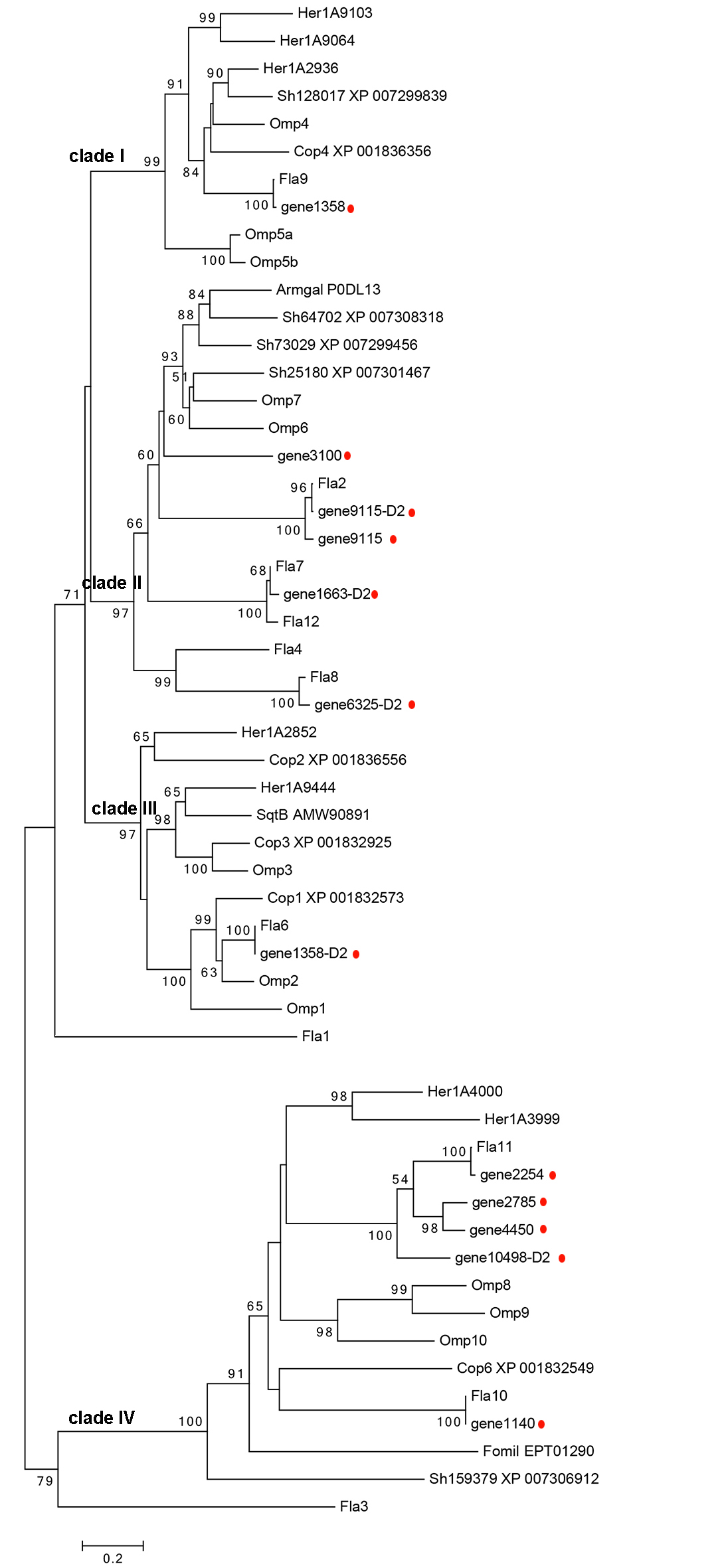

Supplement: Supplementary file 9 — Additional file 9: Fig. S5. Neighbor-Joining phylogram of putative sesquiterpene synthases (STS) of F. filiformis were constructed based homologous protein sequences. The number along branch represent the bootstrap value above 50%. The gene encoding sesquiterpene synthases with red dot were identified in this study. Detail information of the sequences used in phylogram can be found in reference [40, 44]. Labeled in “Cop” from the fungus Coprinopsis cinereus; “Omp” from fungus Omphalotus olearius and “Sh” from fungus Stereum hirsutum. (JPG 439 kb) [file 12864_2020_7108_MOESM9_ESM.jpg]
